# Effect of Chitosan-24-Epibrassinolide Composite Coating on the Quality Attributes of Late-Harvested Pomegranate Fruit under Simulated Commercial Storage Conditions

**DOI:** 10.3390/plants11030351

**Published:** 2022-01-27

**Authors:** Sbulelo Mwelase, Olaniyi Amos Fawole

**Affiliations:** Postharvest Research Laboratory, Department of Botany and Plant Biotechnology, University of Johannesburg, P.O. Box 524, Auckland Park, Johannesburg 2006, South Africa; sbulelom@uj.ac.za

**Keywords:** phytohormone, brassinosteroids, electrolyte leakage, respiration, vitamin C

## Abstract

This study evaluated the efficacy of chitosan (CH) functionalized with 24-epibrassinolide (EBR) coating in terms of preserving the postharvest quality of late-harvested pomegranate (cv. Wonderful) fruit. Late-harvested pomegranate fruit were immersed for 3 min in different surface treatment solutions—CH 1.5% (*w*/*v*), CH + 2 µM EBR, CH + 5 µM EBR, CH + 10 µM EBR and CH + 15 µM EBR—and distilled water was used as a control treatment. The fruit were air-dried and subjected to long storage duration at 5 °C with 85 ± 5 RH for 12 weeks. At 4-week sampling intervals, a batch of fruits was placed at 21 ± 2 °C and 65–70% RH for a further 3 d period to simulate retail conditions before measurements were taken. Fruit physiological responses, physico-chemical properties, phytochemical contents, antioxidant capacity and physiological disorders were monitored during storage. The results showed that the CH-EBR composite edible coatings significantly (*p* < 0.05) delayed degradative processes due to senescence. The CH-EBR treatments delayed colour, texture and total soluble solids (TSS) degradation and reduced weight loss, respiration, electrolyte leakage and spoilage compared to the control and CH treatment. The treatment effect was more noticeable on fruit treated with CH + 10 µM EBR, which exhibited lower weight loss (18.19%), respiration rate (7.72 mL CO_2_ kg^−1^ h^−1^), electrolyte leakage (27.54%) and decay (12.5%), and maintained higher texture (10.8 N) and TSS (17.67 °Brix) compared to the untreated fruit with respective values of 24.32%, 18.06 mL CO_2_ kg^−1^ h^−1^, 43.15%, 37.5%, 8.32 N and 17.03 °Brix. This was largely attributed to the significantly higher antioxidant content, including the ascorbic acid content, total phenol content, total anthocyanin content and DPPH (radical scavenging activity), of the coated fruit compared to the control fruit. Therefore, CH + 10 µM EBR treatment is recommended as a postharvest management strategy to improve the quality preservation of late-harvested pomegranate fruit during storage.

## 1. Introduction

Pomegranate is amongst the most important crops produced globally because of its countless nutritional and medicinal benefits [1]. The functional benefits of pomegranates have resulted in increased consumption and demand for the fruit; consequently, the production and market opportunities have also expanded to meet consumer demand [1,2]. This means that harvest time will also increase due to higher volumes of fruit being harvested. While the global demand for pomegranate is increasing, quality issues remain a concern in most producing regions, leading to substantial postharvest losses and waste [3]. This is a result of the highly perishable nature of pomegranates due to high susceptibility to physiological disorders and decay after harvest. This leads to the overall fruit quality declining, including the loss of functional properties of the fruit.

Cold storage is commonly used to maintain postharvest quality and prolong the storability of fruits. However, pomegranates are highly susceptible to chilling injury (CI) when stored at the recommended refrigeration temperatures, with fungal attacks and husk scald also being postharvest problems encountered during cold storage [4]. The quality, ripening behaviour and susceptibility to physiological disorders of pomegranate are also closely related to harvest maturity [5]. According to Fawole and Opara [5], late-harvested pomegranate fruit have a relatively short postharvest life and tend to develop off-flavours without any visible postharvest physiological disorders if stored for an extended time. Furthermore, Defilippi et al. [6] observed that ‘Wonderful’ pomegranate fruits harvested later in the season were characterized by a higher incidence of decay and scald.

Contrary to Defilippi et al. [6], Kashash et al. [7] reported that early-season ‘Wonderful’ pomegranate fruit were more susceptible to chilling injury and aril browning compared with late-season fruit. This may have resulted from the different molecular mechanisms involved in the development of different physiological disorders. Nonetheless, the high susceptibility to scald and decay in late-harvested pomegranate fruit, as reported by Defilippi et al. [6], suggests the need for postharvest technologies to maintain quality and extend the storage life of the late-harvested pomegranate fruit.

Edible coatings constitute a promising technology that is currently used to extend the storage life and maintain the quality of various minimally processed and whole fruits and vegetables [8,9,10,11,12,13,14,15]. The potential ability of coatings to maintain the quality and extend the postharvest life of horticultural produce is due to the barrier applied for the permeability of gases, mainly CO_2_ and O_2_, which reduces metabolic processes of the fruit and vegetables [16,17,18]. Edible coatings have attracted much interest, mainly because they are derived from natural sources and are, therefore, eco-friendly and biodegradable [19]. Thus, edible coatings provide alternatives to synthetic chemicals and polyethylene packaging bags in postharvest management, which impose an environmental threat. Edible coatings also constitute an ideal carrier of active agents, such as antioxidant, antimicrobial and flavouring compounds, with the advantage of providing improved coating properties for the improved postharvest management of horticultural crops by combining several polymers [20,21].

**Figure 1:**
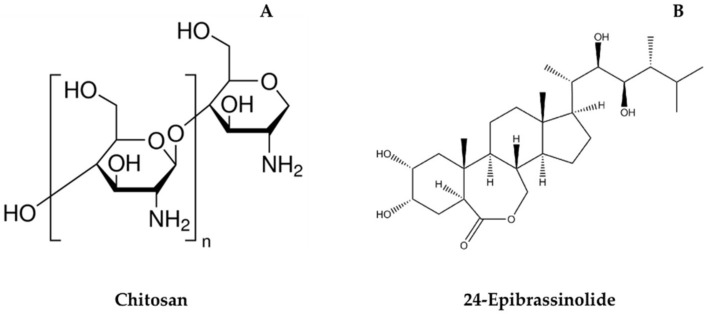
Chemical structures of chitosan (**A**) and 24-Epibrassinolide (**B**).

Chitosan (poly β-(1, 4)-2-amino-2-deoxy-D-glucan) (CH) (Figure 1A) is an exceptional polysaccharide coating material that is widely used in the postharvest quality management of horticultural crops [22,23,24]. The popularity of this biopolymer is attributed to its excellent film-forming properties, biodegradability, nontoxicity and antimicrobial properties [24,25,26]. However, the application of CH is restricted due to its low antioxidant capacity [22]. As with the other coating materials, the nature of CH also allows for the incorporation of different bio-compounds to improve the coating functionality. Hence, our study is focused at the incorporation of CH with brassinosteroids (BRs). 

BR is a natural, nontoxic, non-genotoxic, biosafe and eco-friendly phytohormone that represents an alternative to improve the properties of edible coatings [27]. As a phytohormone, BRs endogenously exists in all plant species at very low concentrations and are integrated in plants’ responses against stress [28,29]. Several BRs have been identified to date; however, among these, brassinolide, 24-epibrassinolide (EBR) and 28-homobrassinolide are, so far, the most biologically active forms, with EBR (Figure 1B) being the most extensively used BR analog in physiological studies [30,31]. This is attributed to the greater bioactivity of EBR compared to the BR active analogs. Recently, the United States (US) and the European Union (EU), the major importing regions, have exempted residue limits of EBR from foods and declared its nontoxicity as a plant growth regulator in agriculture [32,33]. For this reason, several researchers have attempted to develop alternative safe postharvest preservation strategies using BRs for the improved quality management of horticultural crops.

Exogenous postharvest applications of BRs have been reported to enhance bio-active compounds [34,35,36,37], delay ripening and senescence [35], and regulate defence-related enzymic activities, which develop robust defence mechanisms against different physiological disorders [34,35,37,38,39]. The mechanism of BRs further involves (1) the upregulation of membrane protein genes (leading to higher membrane integrity) [29,31], (2) the enhancement of phenylalanine ammonia lyase (PAL) and suppressed polyphenol oxidase (PPO) enzymatic activities, leading to higher phenol and anthocyanins accumulation [39,40,41,42], and (3) higher arginine pathway activity, leading to higher polyamines and proline accumulation [39,43,44], resulting in the improved antioxidant capacity of the crop. Notably, single phytohormone treatment is the main formulation of BRs’ exogenous postharvest applications for the preservation of horticultural crops. While the use of BRs alone has shown satisfactory effects in maintaining the quality and delaying the senescence of fruit and vegetables during storage, its effect cannot be sustained due to the poor adhesion ability of BRs on fruit surfaces. Therefore, incorporating BRs with CH is hypothesized to show synergistic effects resulting in improved coating functionality and effectiveness. Such an effect has been previously reported, but with other phytohormones. Zhang et al. [45] reported that incorporating chitosan (CH) with salicylic acid remarkably preserved the quality and antioxidant capacity of cucumber compared to the use of chitosan and salicylic acid alone during postharvest storage. In addition, Sayyari et al. [46] observed enhanced performance of salicyloyl chitosan treatment on pomegranate fruit compared to the use of salicylic acid and chitosan treatment alone during storage at 2 °C for 5 months. Therefore, the aim of this study was to develop and evaluate the efficacy of CH functionalized with EBR in effectively maintaining the quality and delaying the senescence of late-harvested pomegranate fruit subjected to commercial storage conditions.

## 2. Results and Discussion

### 2.1. Physiological Responses

#### 2.1.1. Weight Loss

**Figure 2:**
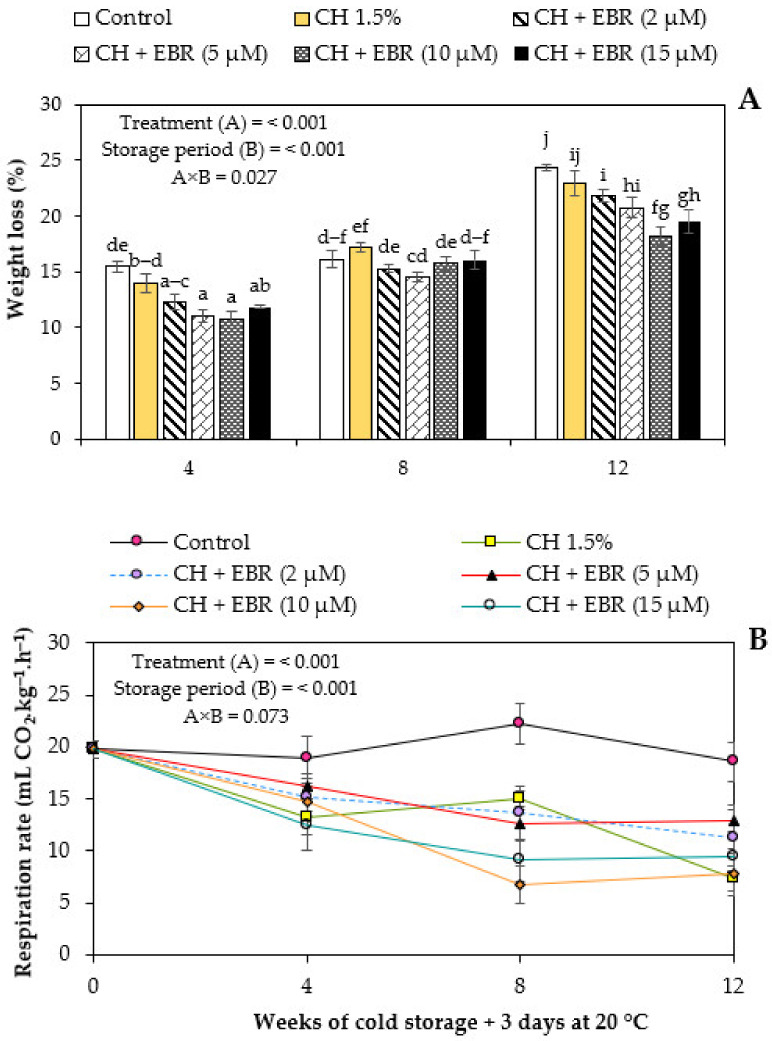
Weight loss (**A**) and respiration rate (**B**) of uncoated (control) and coated pomegranate fruit during storage at 5 °C for 12 weeks plus 3 days at 20 °C. Each bar represents mean ± error bars. Error bars represent standard error (SE) of the mean values, and different letters on bars represent statistical differences (*p* < 0.05) according to Duncan’s multiple range test.

The weight loss of pomegranate fruit increased progressively (*p* < 0.05) during storage regardless of treatment (Figure 2A). The highest weight loss was observed in control fruit and the lowest weight loss was observed in fruit treated with CH + 10 µM, followed by the CH + 15 µM EBR, CH + 5 µM EBR, CH + 2 µM EBR and CH treatments. It was noted that CH in combination with EBR significantly (*p* < 0.05) reduced pomegranate fruit weight loss compared to CH treatment. Similar results were reported by Zhang et al. [45] and Sayyari et al. [46], where CH-incorporated salicylic acid significantly lowered the weight loss of cucumber and pomegranate fruit, respectively, compared with the use of CH and salicylic acid alone. 

Loss of fruit weight is mainly due to water loss caused by transpiration, and edible coatings form a semi-permeable layer on fruit surfaces, which become a protective barrier to reduce transpiration rates [47]. However, this is highly influenced by the nature of the edible coating; for instance, CH is hydrophilic, resulting in poor water vapor barrier properties [24]. This agrees with our results where CH treatment showed reduced efficacy in retarding fruit weight loss during storage. However, it could be hypothesized that incorporating EBR into the coating matrix improved the water vapor barrier properties of the coating through its preventive effect against membrane damage [35], which results in high membrane integrity and, thus, further reduced transpiration rates.

#### 2.1.2. Respiration Rate

The process of respiration is essential and is a good index of fruit quality during postharvest storage. During this process, the stored carbohydrates are broken down using oxygen as a co-substrate to produce the energy (ATP) necessary for the activity of the enzymes involved in metabolic processes. Thus, lower respiration rates are crucial to maintaining quality and extending fresh produce’s postharvest life. According to the factorial analysis, changes in fruit respiration were driven by the combination of treatment (*p* < 0.001) and storage duration (*p* < 0.001) (Figure 2B). 

The rate of respiration of all the treated fruit remained steady and lower than that of the control fruit throughout storage (Figure 2B). The control fruit exhibited an increase in respiration rates and reached a peak at week 8 of storage; inversely, the EBR-treated fruits showed a declining rate with storage. While the CH also exhibited a declining rate, an increase was observed at week 8 of storage. Nevertheless, the peak was significantly lower than that of the control fruit. This was primarily due to the excellent gas permeability properties of CH, resulting in high carbon dioxide and low oxygen within the fruit, thus lowering respiration rates [13]. 

The enhanced effectiveness of CH + EBR coatings, in terms of reducing the respiration rates, compared to CH alone could be attributed to the coating being more efficient in terms of restricting gaseous exchange due to EBR maintaining higher membrane integrity [35]. In agreement with our findings, Zhu et al. [48] and Wang et al. [35] reported reduced respiration rates of EBR-treated Jujube and kiwifruit, respectively, during storage.

#### 2.1.3. Fruit Texture and Aril Hardness

Fruit firmness decreased significantly (*p* < 0.001) in all treatments during storage (Table 1), and both factors (treatments: *p* < 0.001; storage period: *p* < 0.001) contributed to the changes observed in fruit firmness. However, the firmness decreased more slowly in treated fruits, with slight significant differences observed between CH treatment and in combination with EBR bioactive compound during storage. At the end of storage (week 12), CH + EBR (10 µM) treatment showed an optimum effect in retaining fruit firmness compared to all the treatments. In agreement with our findings, Gao et al. [39] and Wang et al. [35] reported higher firmness of EBR-treated peach and kiwifruit, respectively, during storage. The delay in loss of fruit firmness, as influenced by both CH and EBR, may be attributed to the modified fruit gas atmosphere by the coating, which results in reduced activity of cell wall degradation enzymes, including polygalacturonase (PG), pectinmethylesterase (PME), galactosidase and cellulase [46,49]. Reduced activity of these enzymes results in higher levels of pectin, the key substances involved in the mechanical strength of the cell wall [49].

As observed for fruit firmness, aril hardness decreased significantly (*p* < 0.001) with prolonged storage, with significant differences observed amongst coating treatments (*p* < 0.001) (Table 1). The arils’ hardness was also considerably (*p* < 0.05) affected by the coating treatments. The overall decrease in the arils’ textural property during storage shows that CH + EBR treatments significantly lowered the loss of the arils’ textural property compared to the control and CH treatments. Fawole and Opara [5] reported that high aril hardness is partly due to higher juice contents, resulting in higher turgor pressure within the arils. Thus, the efficacy of CH and EBR treatments to effectively reduce transpiration could be attributed to the observed effect on aril hardness. CH + EBR (10 µM) treatment showed a good effect in terms of reducing the loss of aril hardness, and delayed senescence better compared to the other treatments.

#### 2.1.4. Electrolyte Leakage

Electrolyte leakage (EL) is generally accepted as an index that quantifies membrane damage and the loss of the membrane integrity of fresh produce [50]. The damage is mediated by increased production of H_2_O_2_ and the bursting of other reactive oxygen species (ROS), and thus, increasing plant oxidative stress causes the leakage of membrane intracellular ions and metabolites [51]. The EL of control and treated pomegranate fruit during storage is shown in Table 1. The EL of pomegranate fruit increased with storage in all treatments; however, EBR treatments significantly (*p* < 0.001) inhibited the leakage increase (Table 1). The highest EL was observed in the control fruit, followed by CH treatment, with CH + EBR (5 µM) showing the optimum effect in terms of limiting leakage ions and membrane damage. 

The suppression of EL by EBR treatments was also reported by Aghdam and Mohammadkhani [37] on tomato, Pakkish et al. [42] on grapes and Wang et al. [35] on kiwifruit. The literature suggests that the EBR hormone alleviates membrane damage by regulating the activity of antioxidant defence systems, which inhibit the burst in accumulation of toxic ROS. Ghorbani and Pakkish [41] and Pakkish et al. [42] observed higher catalase (CAT) activity in BR-treated orange (1.5 ppm) and grapes (10 µM), respectively. Aghdam et al. [40] noted that postharvest EBR treatment enhanced the PAL activity of tomato fruit, which was significantly higher than in control fruit, an effect associated with higher membrane integrity and lower EL.

### 2.2. Physico-Chemical Properties

#### 2.2.1. Colour Attributes

**Figure 3:**
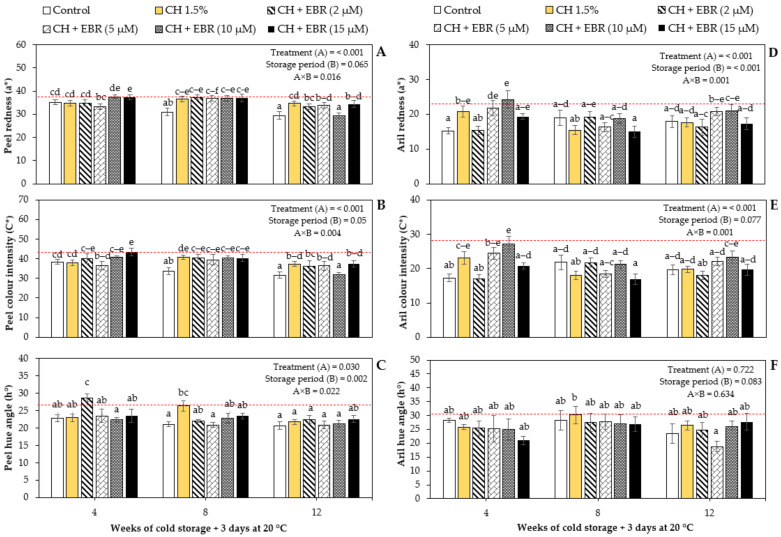
Changes in colour parameters; (**A**) peel redness (a*), (**B**) peel chroma (C*), (**C**) peel hue angle (h°), (**D**) aril redness (a*), (**E**) aril chroma (C*) and (**F**) aril hue angle (h°) of pomegranate fruit during storage at 5 °C for 12 weeks plus 3 days at 20 °C. Each bar represents mean and error bars. Error bars represent SE of the mean values, and different letters on bars represent statistical differences (*p* < 0.05) according to Duncan’s multiple range test. Dotted lines represent measurements at harvest.

Colour is an important parameter that plays a significant role in the acceptability of foods. The colour changes of pomegranate peel and arils, as determined by CIELAB values (a*: redness; C*: chroma (colour intensity); h°: hue angle), are presented in Figure 3A–F. There was a significant interaction between the main factors for all the investigated colour attributes except for the aril hue angle. Noticeably, coated fruit retained higher peel redness, as indicated by a* values throughout storage, compared with control fruit, which showed the lowest values of a*. However, during the last week of storage (week 12), significantly lower peel redness was observed for CH + EBR (10 µM)-treated fruits. Colour intensity decreased gradually with storage and was also highly affected (*p* < 0.001) by the coating treatments (Figure 3B). A similar effect was observed where the coating treatments maintained significantly (*p* < 0.05) higher chroma values throughout the storage period as compared to the control treatment. In agreement with our findings, Fawole and Opara [1] reported decreasing peel redness and colour intensity over the storage period. The hue angle behaviour was unclear; nonetheless, insignificant differences were observed between most treatments on different sampling days (Figure 3C).

With regard to the aril colour appearance, there were inconsistent fluctuations during storage; however, the overall trend showed that aril redness decreased significantly (*p* < 0.001) with storage (Figure 3D). However, storage period did not have a significant effect (*p* = 0.077) on the colour intensity of the arils (Figure 3E). This is in line with the report by Fawole and Opara [1], who observed non-significant changes in the aril colour intensity of ‘Bhagwa’ pomegranate cultivar during storage. It was suggested that pomegranate’s aril colour is relatively stable during storage. Neither the storage period nor the coating treatments had a significant effect on the hue angle (treatments: *p* < 0.722; storage period: *p* < 0.083), which suggests that the arils’ colour saturation remained stable during storage. Notably, CH + EBR (10 µM) treatment maintained higher values of redness and chroma. The retention of the colours of arils from fruit subjected to CH + EBR (10 µM) treatment could be attributed to the ability of the coating to reduce the activity of enzymes associated with the degradation of anthocyanins in pomegranate fruit. 

#### 2.2.2. Titratable Acidity, Total Soluble Solids and BrimA

The changes in the total soluble solids (TSS) and titratable acidity (TA) of treated and control pomegranate fruits during storage are presented in Table 2. These two attributes are important in determining the taste quality of pomegranate fruit. In this study, TA decreased significantly (*p* = 0.011) with prolonged storage. As shown in Table 1, the overall effect of coating treatments on TA was not significant (*p* = 0.279). However, control fruit were characterized by lower TA from week 8 of storage until the end of the experiment as compared to the treated fruits, which showed the highest TA. On the last day of storage, the TA values in fruit treated with CH, CH + EBR (2 µM), CH + EBR (5 µM), CH + EBR (10 µM) and CH + EBR (15 µM) were 0.85, 0.97, 0.96, 0.93 and 1% citric acid, respectively, which were all higher than the control values (0.81% citric acid). The literature has shown that TA is directly correlated with the concentration of organic acids in the fruit, which are the main respiratory substrates in pomegranates [52]. Thus, the gradual decline in TA with storage indicates the ongoing metabolic processes in the fruit during storage. Therefore, higher TA during storage indicates a prolonged storage life and high quality of the fruit. The high TA in the coated fruits could result from the enhanced effectiveness of the coatings in terms of reducing respiration rates, as previously explained. The lower respiration rates result in fewer organic acids being used up.

The TSS also decreased gradually with storage (Table 2). Our findings agree with those of Fawole and Opara [1], who reported a decrease in TSS content in ‘Ruby’ pomegranate fruit with an increased storage period. TSS are sugars that are also used as an energy source in pomegranate for respiration [53]. This helps to explain the declining TSS content with storage. Notably, the coating treatments maintained significantly (*p* < 0.001) higher levels of TSS than the control during storage. At the same time, there were slightly significant differences between CH coating alone and in combination with EBR. During the last week of storage, the highest level of TSS (17.67 °Brix) was recorded in fruits coated with CH + EBR (10 µM) and the lowest levels (17.03 °Brix) were observed in control fruits. The BrimA index did not change significantly (*p* > 0.131) with storage (Table 1). Contrary to our results, Fawole and Opara [5] and Fawole et al. [17] reported a decrease in the BrimA index for ‘Bhagwa’ and ‘Wonderful’ pomegranate fruit with prolonged storage durations. Arendse et al. [54] observed an increase in the BrimA index of ‘Wonderful’ pomegranate fruit during storage. Notably, the BrimA index was influenced by the coating treatments (*p* = 0.038) and, overall, treating fruit with CH + EBR (10 µM) resulted in a better BrimA index compared to other treatments.

### 2.3. Phytochemical Content and Antioxidant Properties

#### 2.3.1. Ascorbic Acid Content

**Figure 4:**
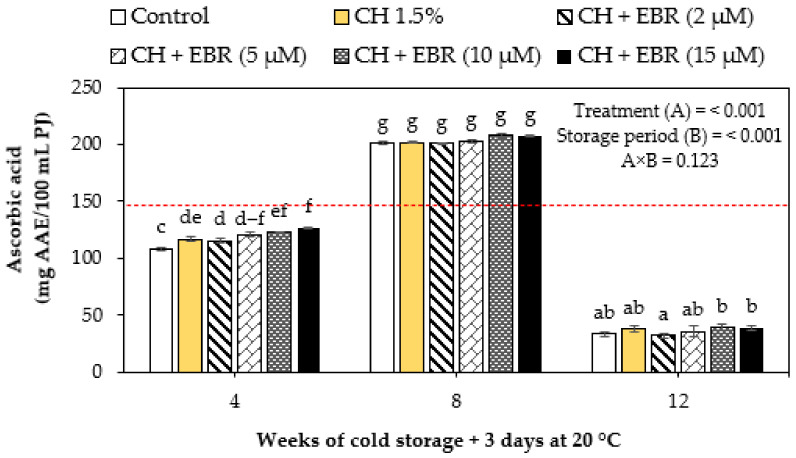
Ascorbic acid content of uncoated (control) and coated pomegranate during storage at 5 °C for 12 weeks plus 3 days at 20 °C. Each bar represents mean ± error bars. Error bars represent SE of the mean values, and different letters on bars represent statistical differences (*p* < 0.05) according to Duncan’s multiple range test. Dotted line represent measurement at harvest.

Generally, the changes in ascorbic acid content were influenced by the combined effects of treatment (*p* < 0.001) and storage period (*p* < 0.001). The ascorbic acid content initially declined from harvest to week 4 of storage, thereafter peaked at week 8 of storage, and then declined. Thus, overall, the ascorbic acid contents of pomegranate fruit decreased with storage. Noticeably, the ascorbic acid levels were higher in the treated fruit and lower in the control fruit (Figure 4). At the end of storage (week 12), the ascorbic acid content of the control fruit was 33.28 mg AA/100 mL. For the CH, CH + EBR (2 µM), CH + EBR (5 µM), CH + EBR (10 µM) and CH + EBR (15 µM) treatments, the ascorbic acid content reached 37.79, 33.79, 35.77, 39.04 and 38.63 mg AA/100 mL, respectively, which were all significantly higher than control. Ascorbic acid is reported to be the most important water-soluble antioxidant that directly protects the plant against oxidative stress [55].

Further, a decrease in ascorbic acid content was found to result from the enzyme activity of ascorbate oxidase (AO) using oxygen as the primary substrate, which caused degradation in ascorbic acid [55]. Thus, the excellent gas barrier properties of CH resulted in lower O_2_ within the fruit and consequently lower oxygen availability for AO activity. The high ascorbic acid content in fruit treated with CH + EBR (10 µM) and CH + EBR (15 µM), respectively, could be attributed to the direct involvement of EBR in detoxifying ROS in cells [35]. Ascorbic acid breaks down H_2_O_2_ to water [35], an effect associated with reduced fruit susceptibility to physiological disorders, delayed senescence, and high-quality maintenance.

#### 2.3.2. Total Phenolic Content

**Figure 5:**
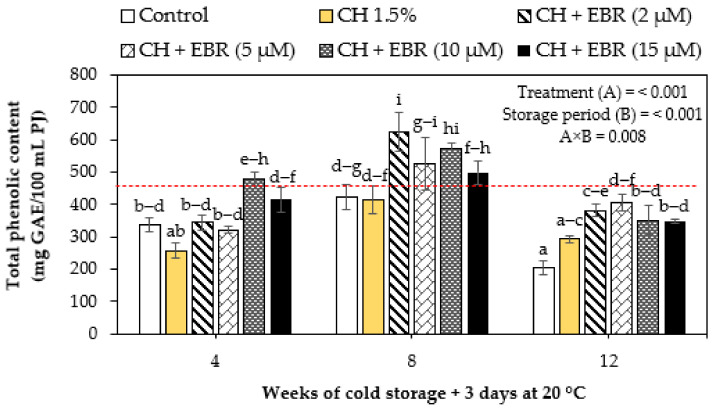
Total phenolic content of uncoated (control) and coated pomegranate during storage for 12 weeks at 5 °C plus 3 days at 20 °C. Each bar represents mean ± error bars. Error bars represent SE of the mean values, and different letters on bars represent statistical differences (*p* < 0.05) according to Duncan’s multiple range test. Dotted line represent measurement at harvest.

Changes in the total phenolic content (TPC) of coated and control pomegranate fruit during storage are presented in Figure 5. Generally, the TPC significantly (*p* < 0.001) decreased with storage. However, significant enhancements were observed at week 8 of storage but thereafter declined to the end of storage. Our findings agree with those of Fawole and Opara [1], who reported a decline in TPC in ‘Ruby’ and ‘Bhagwa’ pomegranate fruit during storage at 5 °C for 16 weeks. Similarly, Sayyari et al. [30] reported a decline in TPC in pomegranate fruit (cv. Malase) coated with 1–2% CH and stored at 4.5 °C for 120 days. Noticeably, the coating treatments significantly suppressed (*p* < 0.001) the decline in TPC during storage. At the end of the experiment (week 12), fruits coated with chitosan + EBR (5 µM) significantly maintained a higher TPC than other treatments.

The changes in TPC during cold storage may be related to fluctuations in the PAL and PPO enzyme activities [56]. PAL is the key enzyme in the phenylpropanoid pathway that is directly involved in the biosynthesis of phenolic compounds. In contrast, PPO activity results in the oxidation of phenolic substances using oxygen as a co-substrate. The consequently lower oxygen within coated fruit could be attributed to the higher TPC in treated fruit due to reduced PPO activity. The inhibition of PPO activity by coating treatments has been largely observed in the literature [57,58,59,60]. Furthermore, BRs have been reported to induce higher PAL activity [40,46], which is integrated with the BR-induced plant defence mechanism. Therefore, the lower PPO activity and higher PAL activity in CH + EBR-coated pomegranate resulted in the observed higher TPC.

#### 2.3.3. Total Anthocyanin Content

**Figure 6:**
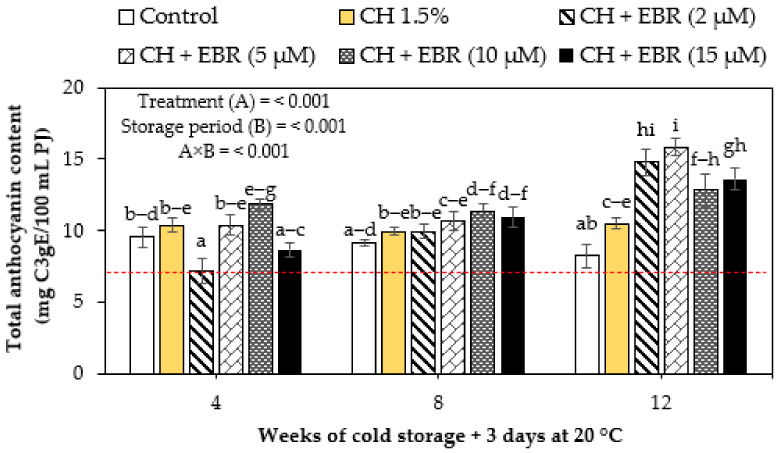
Total anthocyanin content of uncoated (control) and coated pomegranate during storage for 12 weeks at 5 °C plus 3 days at 20 °C. Each bar represents mean ± error bars. Error bars represent SE of the mean values, and different letters on bars represent statistical differences (*p* < 0.05) according to Duncan’s multiple range test. Dotted line represent measurement at harvest.

Total anthocyanin content (TAC) changes in coated and uncoated (control) pomegranate fruit during storage are presented in Figure 6. TAC significantly increased with storage in the coated fruit. In contrast, the TAC significantly declined in the uncoated fruit with storage. Thus, significantly lower TAC was observed in the uncoated fruit on the last week of storage, followed by the CH treatment with CH + EBR (5 µM) treatment showing high TAC. Anthocyanins are regarded as non-enzymatic antioxidants in plants, suggesting that the EBR-mediated accumulation in anthocyanins is part of the mechanisms employed to maintain the quality of pomegranate fruit. As previously explained, the induced higher PAL enzyme activity and suppressed PPO activity in EBR-treated fruit is suggested to have resulted in higher TAC. In agreement with our findings, Sayyari et al. [46] reported an enhanced accumulation of TAC in ‘Mallas Saveh’ pomegranate fruit coated with salicylic acid + chitosan during storage at 2 °C for 5 months.

#### 2.3.4. DPPH Radical-Scavenging Activity

**Figure 7:**
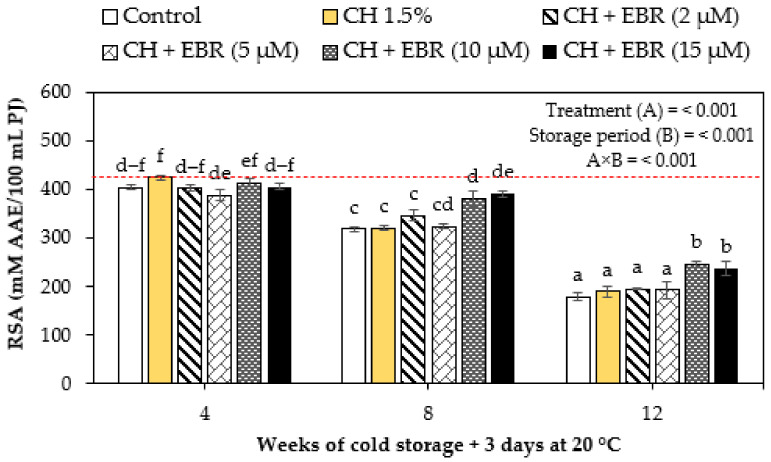
DPPH radical-scavenging activity of uncoated (control) and coated pomegranate fruit stored for 12 weeks at 5 °C plus 3 days at 20 °C. Each bar represents mean ± error bars. Error bars represent SE of the mean values s, and different letters on bars represent statistical differences (*p* < 0.05) according to Duncan’s multiple range test. Dotted line represent measurement at harvest.

The radical-scavenging activity of pomegranate fruit significantly (*p* < 0.05) declined gradually with storage in all the treatments (Figure 7). The coating treatments also significantly (*p* < 0.05) affected the radical-scavenging activity of pomegranate fruit. However, insignificant differences (*p* > 0.05) were observed between control, CH alone, and CH in combination with EBR at 2 and 5 µM treatments at week 8 and week 12 of storage. Further, the treatments of CH + EBR (10 µM) and CH + EBR (15 µM) were statistically similar during the last week of storage. However, these treatments maintained higher radical-scavenging activity throughout the storage period of the fruit. The higher radical-scavenging activity of CH + EBR-treated fruit may be attributed to the enhanced antioxidant enzyme activity induced by EBR, thereby improving the fruit’s overall antioxidant capacity and scavenging ability [61]. In support of our findings, previous studies have demonstrated enhanced CAT, superoxide dismutase (SOD) and ascorbate peroxidase (APX) antioxidant enzymes activities in mandarins [62], mushrooms [63] and grapefruits [42] treated with EBR. The authors further reported that enhanced antioxidant enzyme activities were correlated with reduced H_2_O_2_ production, confirming the improved radical-scavenging ability of treated fruit.

#### 2.3.5. Ferric Reducing Antioxidant Power (FRAP)

**Figure 8:**
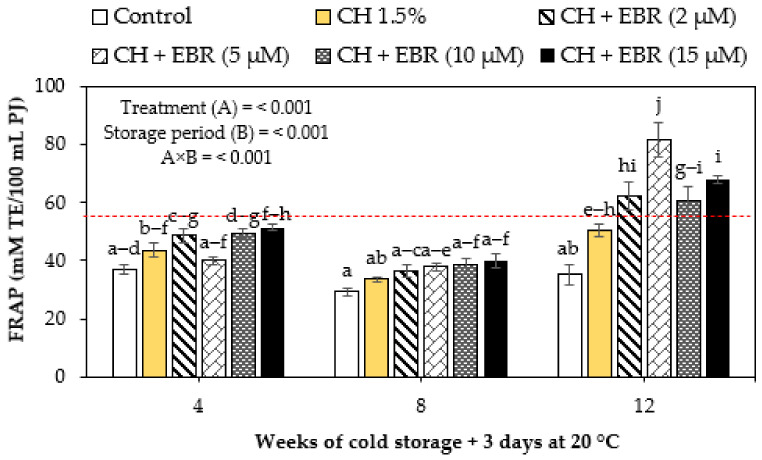
Ferric reducing antioxidant power (FRAP) of uncoated (control) and coated pomegranate fruit stored for 12 weeks at 5 °C plus 3 days at 20 °C. Each bar represents mean ± error bars. Error bars represent SE of the mean values, and different letters on bars represent statistical differences (*p* < 0.05) according to Duncan’s multiple range test. Dotted line represent measurement at harvest.

Generally, the changes in ascorbic acid content were influenced by the combined effects of changes in the FRAP of uncoated and coated pomegranate fruits during storage, as presented in Figure 8. The FRAP assay has been widely applied as an index of the total antioxidant content of foods. In our study, the FRAP of both coated and uncoated pomegranate fruit significantly declined during storage up to week 8, which thereafter increased. The changes in the antioxidant power of pomegranate fruit were highly influenced by the interaction between the main factors investigated (*p* < 0.001). The coated fruit showed significantly (*p* < 0.001) higher antioxidant power throughout storage than the control fruit. The reason for the later increase in the antioxidant power of pomegranate fruit remains unclear; however, it can result from the induced accumulation of anthocyanins and enhanced antioxidant enzyme activities, thereby improving the fruit’s overall antioxidant content. On the contrary, Fawole and Opara [1] reported an early increase in FRAP after 4 weeks of storage, which then declined for the rest of the storage period. 

### 2.4. Physiological Disorders

**Figure 9:**
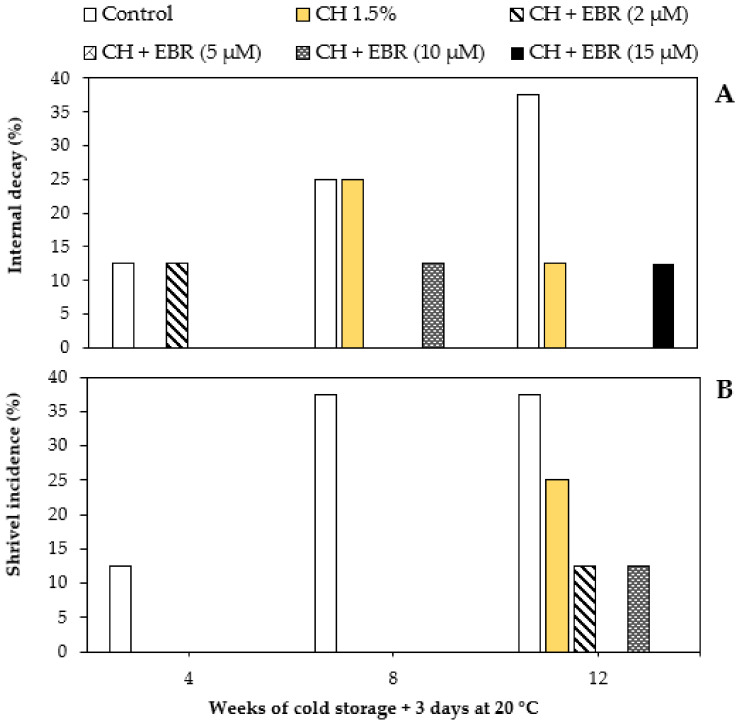
Internal decay (**A**) and shrivel incidence (**B**) of uncoated (control) and coated pomegranate fruit stored for 12 weeks at 5 °C plus 3 days at 20 °C.

**Figure 10:**
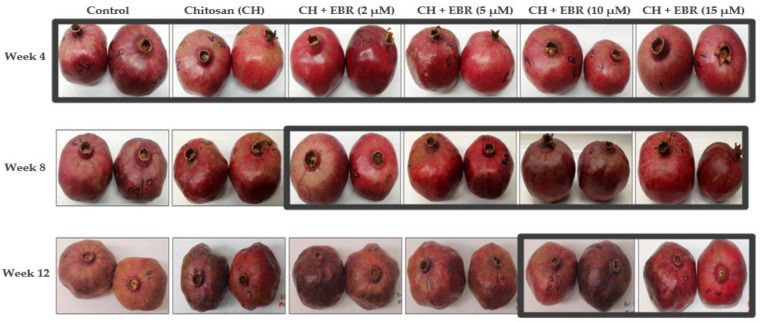
Visual appearances of uncoated (control) and coated pomegranate fruit during storage at 5 °C for 12 weeks plus 3 days at 20 °C.

The internal decay in the late-harvested pomegranate fruit was attributed mainly to aril browning, while the external decay resulted from shrivelling (Figure 9A,B). The incidence of both the physiological disorders increased with storage in the control fruit; however, inconsistent changes were observed in the coated fruit. In support of our findings, Fawole et al. [17] reported increased severity and incidence of the internal decay of ‘Wonderful’ pomegranate fruit with extended storage. The reduction in physiological disorder incidence and susceptibility by the EBR treatment might be attributed to the stimulated activity of antioxidant enzymes, which results in enhanced antioxidant capacity of fruit and, in turn, reduced ROS production, which mainly results in physiological disorders. The visual appearances of fruit during storage showing the marked increase in shrivelling are shown in Figure 10. The appearances showed an increase in shrivelling disorder with storage. The apparent increases were observed in the control and CH 1.5% treatments, which showed significantly higher shrivelling after 8 weeks of storage. Further, the CH + EBR (2 µM) and CH + EBR (5 µM) treatments showed a high increment in shrivelling after 12 weeks of storage, whereas the score of the other treatments was still lower. This suggests that the CH + EBR (10 µM) and CH + EBR (15 µM) treatments significantly inhibited shrivelling and better maintained the visual appearance of fruit until the last week of storage.

### 2.5. Correlation Matrix and Principal Component Analysis

Pearson’s correlation was used to reveal the degree of correlation between selected variables during the last week of storage (Table 3). Weight loss was negatively correlated with fruit texture (r = −0.87) and aril hardness (r = −0.94). This relationship clearly showed that an increase in fruit weight loss decreased fruit texture and aril hardness, which indicates senescence. A strong and significant negative correlation was recorded for fruit texture and electrolyte leakage (r = −0.86), supporting our previous discussion that increased leakage of intracellular ions and metabolites results in reduced membrane integrity. Furthermore, electrolyte leakage was negatively and strongly correlated with TPC (r = −0.97), TAC (r = −0.95) and FRAP (r = −0.95), suggesting that enhancements in the antioxidant capacity of the fruit, induced by CH + EBR coatings, inhibited the leakage of intracellular ions. Moreover, this suggests that total phenolics, including anthocyanins, and FRAP mainly influenced the fruit’s antioxidant capacity since ascorbic acid and DPPH showed a poor correlation to electrolyte leakage. This was further supported by the observed significant strong negative correlation of internal decay with TPC (r = −0.94) and TAC (r = −0.87). In addition, shrivelling disorder was negatively correlated with TPC (r = −0.9), TAC (r = −0.92) and FRAP (r = −0.96). Moreover, TA was positively and strongly correlated with TPC (r = 0.88), TAC (r = 0.91) and FRAP (r = 0.86), suggesting that limiting the consumption of organic acids through respiration, and hence, delayed senescence, could be attributed to high antioxidant systems in the fruit, as evidenced by the high TPC, TAC and FRAP in the treated fruit. Significant positive correlations were recorded for TAC and TPC (r = 0.98), FRAP and TPC (r = 0.94), and FRAP and DPPH (r = 0.95).

**Figure 11:**
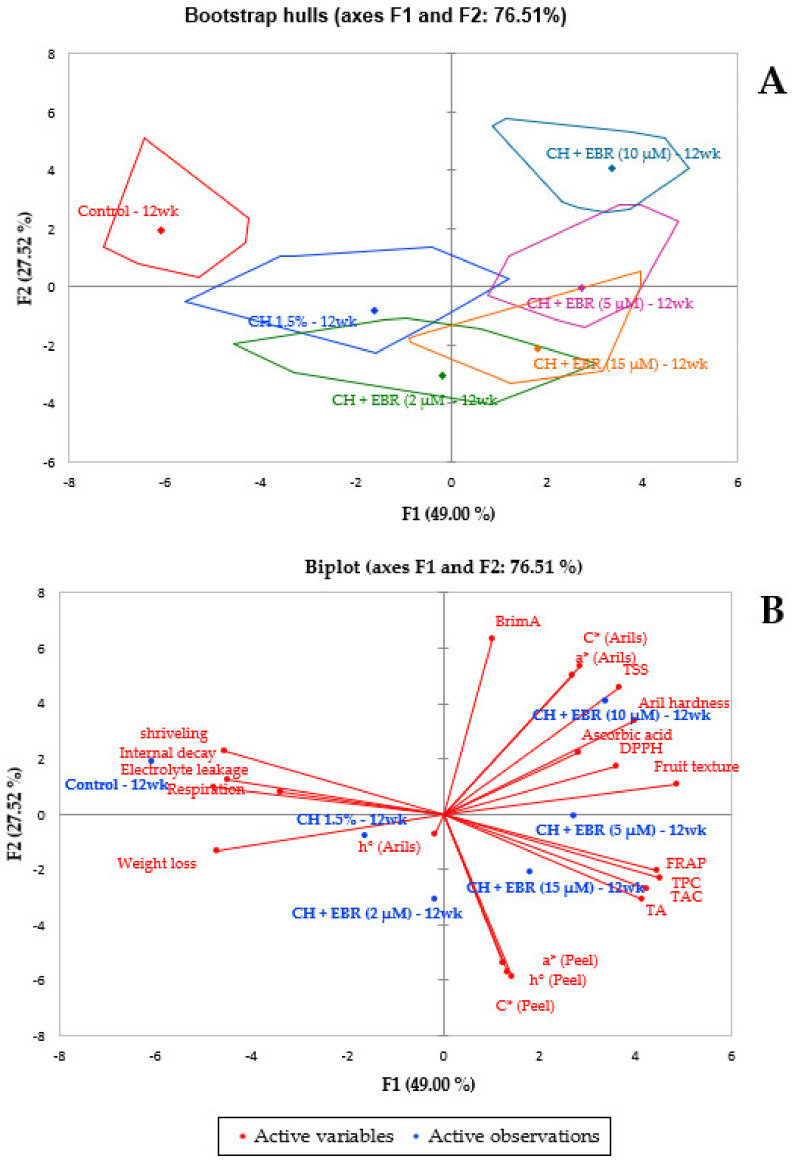
Principal component analysis (PCA) biplot showing the correlation between the measured parameters and coating treatment during the last week (week 12) of storage. Bootstrap hulls (**A**) and biplot (**B**). (a*) redness, (C*) colour intensity, (h°) hue angle, (TA) titratable acidity, (TSS) total soluble solids, (TAC) total anthocyanin content, (TPC) total phenolic content, (DPPH) 2,2-diphenyl-1-picryl hydrazyl and (FRAP) ferric ion reducing antioxidant power.

A principal component analysis (PCA) bootstrap hulls and PC×iplot were generated to demonstrate distinctness between the coating treatments based on the evaluated parameters on the last week of storage (Figure 11A,B). The first and second components explained 49% and 27.52% of the variation, respectively. Thus, the studied principal components explained 76.51% of the total variation. From the bootstrap hulls plot (Figure 9A), six detectable groupings were evident, which include control, CH 1.5%, CH + 2 µM EBR, CH + 5 µM EBR, CH + 10 µM EBR and CH + 15 µM EBR. A PCA biplot was used to identify the features responsible for the clustering patterns (Figure 11B). Cluster 1 (Control) was characterized by higher respiration, electrolyte leakage, decay and shrivelling. Cluster 2 (CH 1.5%) was closely related to cluster 1 and was characterized by high weight loss. Cluster 3 (CH + 2 µM EBR) was characterized by high colour intensity and redness of the peel. Cluster 4 (CH + 15 µM EBR) was characterized by high TA, TAC, TPC and FRAP. These parameters were negatively correlated with the parameters in cluster 1. Cluster 5 (CH + 5 µM EBR) was a crosslink between cluster 4 and cluster 6 and did not have individual characterization. Cluster 6 (CH + 10 µM EBR) was characterized by high colour intensity of the arils, TSS, aril hardness, ascorbic acid, DPPH and fruit texture.

## 3. Materials and Methods

### 3.1. Procurement and Handling of Fruit

Mature pomegranate fruit (cv. Wonderful) were procured from Ubali Pomegranate Farm, Kameelfontein, Johannesburg (25°38′31.9″ S, 28°27′33.0″ E), South Africa, during the 2020/2021 growing season. The fruit were immediately transported in a well-ventilated vehicle to the postharvest and agroprocessing research laboratory at the University of Johannesburg (PARL-UJ). Upon arrival, fruit were sorted for uniformity of colour and size, and fruit with blemishes and external damage were removed. The fruit were then disinfected with 0.01% sodium hypochlorite and air-dried before treatment application.

### 3.2. Preparation of Treatments

The polysaccharide-based edible coating, based on chitosan (Sigma Aldrich Chemical Co., St. Louis, MO, USA) at 1.5% (*w*/*v*), was prepared by dissolving 15 g of chitosan in deionized water containing 1% (*v*/*v*) of acetic acid and concentrations of 24-epibrassinolide (EBR) (Sigma Aldrich Chemical Co. St. Louis, MO, USA) at 2, 5, 10 and 15 µM. EBR concentrations were prepared by first dissolving the EBR powder in ethanol and then made to the desired volume with distilled water. The final concentration of ethanol in the solution was about 0.1%. The chitosan-EBR solution was made up to 1 L after adding Tween-80 (0.1% *w*/*v*), glycerol (1% *w*/*v*) and canola oil (1% *w*/*v*). The mixture was further subjected to magnetic stirring for 90 min at a controlled temperature of 40–50 °C to ensure complete solubility. 

### 3.3. Experimental Design

A completely randomized design was used with five boxes as replicates per treatment and each box containing ten randomly selected fruits. Coatings were applied by fruit immersion for 3 min. Distilled water was used as a control treatment. Subsequently, fruits were left to dry at 21 ± 2 °C and 55 ± 5% RH and packed inside standard open-top commercially used cartons. Fruits were then stored at 5 °C with 85 ± 5 RH for 12 weeks. The temperature (°C) and relative humidity (% RH) within the cold room were monitored throughout the storage period using an Ebro EBI300 data logger (Ebro Electronic™, EBI300 data logger, Breisgau, Germany). At each sampling point, a batch of fruit was placed at 21 ± 2 °C and 65–70% RH for a further 3-day period to simulate retail conditions before measurements were made.

### 3.4. Physiological Responses

#### 3.4.1. Weight Loss

Ten randomly selected fruit per treatment were individually weighed at 4-week intervals using an electronic weighing scale (RADWAG Electronic PS 4500.R2.M, Poland, 0.01 accuracy). The results were expressed as mass loss percentages based on the initial fruit mass on the day of harvest [1].

W (%) = [ (Wi − Wf/Wf) × 100](1)

where W is the weight loss (%) of fruit, Wi (g) is the weight of fruit at the day of harvest and Wf (g) is the weight of fruit at the given sampling interval, and the results were expressed as mean ± S.E.

#### 3.4.2. Respiration Rate

The fruit respiration rate was measured using the closed system method previously described by Fawole and Opara [1]. Briefly, in five replicates, two fruit were placed in a 3 L glass jar, hermetically sealed with a lid containing a rubber septum in the middle for 2 hrs at room temperature. After the incubation time, CO_2_ production inside the glass jar was measured using an infrared O_2_/CO_2_ gas analyser (Checkmate 3, PBI Dansensor, Denmark). The results obtained for CO_2_ production were presented as mean ± S.E. (ml CO_2_/kg∙·h).

#### 3.4.3. Electrolyte Leakage

Electrolyte leakage (EL) was determined using the method described by Mirdehghan et al. [64], with modification by Sayyari et al. [65]. Briefly, 6 discs (10 mm) of peel tissue per replicate were cut using a cork borer and then immersed in 25 mL of 0.4 M mannitol and incubated for 4 h at room temperature under constant shaking; subsequently, the initial conductivity (Ci) of the solution was measured using a conductivity meter (Hanna Instruments 9033, Woonsocket, RI, USA). After readings were taken, the samples were autoclaved (Medsource TC-459 autoclave, Taichung, Taiwan) at 101.3 KPa and 121 °C for 20 min and allowed to cool down at room temperature for 24 h before the final conductance (Cf) was measured. EL was expressed as a percentage of total electrical conductivity using Equation (2), according to Ehteshami et al. [12]

EL (%) = [(Ci/Cf) × 100](2)

#### 3.4.4. Textural Dynamics

Fruit texture (puncture resistance) was determined following a procedure developed by Fawole and Opara [66]. This was conducted using a firmness analyser with a 5 mm diameter cylindrical probe (GÜSS-FTA, Strand, South Africa) programmed to penetrate 8.9 mm into the fruit at the speed of 10 mm.s^−1^. Tests were performed on the two equilateral regions of 10 individual fruit. The peak force required to puncture the fruit skin was taken as the puncture resistance, and the results were expressed as mean ± S.E.

The aril compression test was performed using a texture analyser meter (Agrosta texture analyser, Calib, France) with a 35 mm compression probe. A total of 20 arils per treatment were used, and the test was run per aril aligned horizontally on the compression platform. The textural profile was interpreted using force (N) and distance (mm) as the fundamental variables. The instrument’s operating conditions were: 1 mm/s probe test speed and 0.30 N trigger force.

### 3.5. Physico-Chemical Properties

#### 3.5.1. Colour Attributes

Colour measurements were assessed in CIELAB coordinates (L*, a*, b*) using a colourimeter (Konica Minolta Chroma Meter CR-400, Osaka, Japan), which was calibrated using white standard tile. External colour change was monitored by measuring peel colour of eight randomly selected fruits per treatment at the two opposite equatorial positions. Duplicate colour measurements were made on arils placed in a colourless glass Petri dish for internal colour monitoring. The colour parameters, chroma (C*) and hue angle (h°) were then calculated from the (L*, a*, b*) coordinates using the Equations (3) and (4), respectively [1].

C* = [ (a*^2^ + b*^2^)]^1/2^ (3)

h° = arctan (b*/a*)(4)

#### 3.5.2. Total Soluble Solids and Titratable Acidity

Total soluble solids (TSS) and titratable acidity (TA) were estimated from pomegranate juice extracted from the arils of ten fruits per treatment per sampling point. Juice from arils of each fruit was extracted using a juice extractor (Salton juice extractor, Brunswick, Canada) without crushing the seeds. TA was measured using a non-automated laboratory titrator (Thermo scientific, Orion star T910, Chelmsford, MA, USA) according to the method described by Fawole and Opara [1]. Briefly, 2 mL of fresh was mixed with 90 mL of distilled water and titrated with 0.1 M NaOH to a pH value of 8.2. The results were expressed as a percentage of citric acid. TSS was determined using a digital refractometer (Atago, Tokyo, Japan). BrimA, a criterion for consumer acceptance of juice, was also calculated using Equation (5).

BrimA = TSS − *k* (TA) (5)

where *k* is a constant that reflects the tongue’s higher sensitivity index, ranging from 2 to 10. In this study *k*-value of 2 was used to avoid a negative BrimA index of the pomegranate [67].

### 3.6. Phytochemical Contents and Antioxidant Properties

#### 3.6.1. Ascorbic Acid Content

Ascorbic acid content was determined using a method described by Kawhena et al. [14]. Briefly, 1 mL of pomegranate juice (PJ) was extracted with 9 mL of 1% metaphosphoric acid (MPA). The mixture was then vortexed and sonicated (Labotech Sonic Clean; model: 705; Gauteng, South Africa) for 3 min in ice-cold water. The mixture was centrifuged (Heraeus Biofuge, Stratos) at 4 °C for 10 min at the speed of 10,000 rpm. A quantity of 500 µL of the extract was then mixed with 4.5 mL of 2,6-dichlorophenolindophenol (dye) in a dark room. The samples were further incubated for 30 min in the dark. After incubation, the absorbance was measured at 515 nm against a blank in a UV-visible spectrophotometer (United scientific, SP-UV 300, Shanghai, China). The ascorbic acid content of each sample was calculated based on the calibration curve of standard L-ascorbic acid. The results were expressed as milligrams of ascorbic acid equivalent per hundred millilitres of crude pomegranate juice (mg AA/100 mL PJ). 

#### 3.6.2. Total Phenolic Content

Total phenolic compound extraction and quantification was carried out using a Folin andCiocalteu’s method previously described by Fawole and Opara [67] with slight modifications. Briefly, 1 mL of pomegranate juice (PJ) was extracted with 29 mL of 50% aqueous methanol. The resulting mixture was vortexed and then sonicated in ice for 20 min in a cold water bath. In duplicates, 50 µL of methanolic PJ extract was mixed with 450 µL of 50% methanol followed by the addition of 500 µL of Folin and Ciocalteu′s phenol reagent and, after 2 min, sodium carbonate (2%) was added. The mixture was vortexed and incubated for 40 min in a dark room. The absorbance was subsequently measured at 725 nm using a UV–visible spectrophotometer (United scientific, SP-UV 300, Shanghai, China). The results were expressed as milligrams of gallic acid equivalent per 100 mL of crude PJ (mg GAE/100 mL PJ) and expressed as mean ± S.E.

#### 3.6.3. Total Anthocyanin Content

The pH differential method was used to determine the total anthocyanin content using two buffer systems, namely potassium chloride (KCL) buffer for pH 1 and sodium acetate (C_2_H_3_NaO_2_) buffer for pH 4.5. Briefly, 0.5 µL of the extracted juice was mixed with 3.5 mL of KCL and C_2_H_3_NaO_2_ buffers, respectively, and incubated for 30 min in a dark environment. Samples were read at 510 nm and 700 nm in a UV–visible spectrophotometer (United scientific, SP-UV 300, Shanghai, China). The results were expressed as milligram cyanidin-3-glucoside equivalent per 100 mL of crude PJ (mgC3gE/100 mL PJ) according to the following Equations (6) and (7):

A = [(A510 − A700) pH 1 − (A510 − A700) pH 4.5](6)

TAC (mg/100 mL) = [(A × MW × DF × 100/ϵ] × L(7)

where A = Absorbance values at 510 nm and 700 nm, ϵ = Cyanidin-3-glucoside molar absorbance (26,900), MW = Cyanidin-3-glucoside molecular weight (449.2 g/mol), DF = Dilution factor, L = Cell path length (1 cm) and TAC = total anthocyanin content.

#### 3.6.4. DPPH Radical-Scavenging Activity

The DPPH assay was carried out according to the method reported by Fawole and Opara [67]. Methanolic extract of PJ (15 µL) was then diluted with 735 µL methanol followed by the addition of 750 µL methanolic DPPH solution (0.1 mM). The mixture was vortexed and incubated for 30 min in the dark. Thereafter, the absorbance was measured at 517 nm using a UV–vis spectrophotometer (United scientific, SP-UV 300, Shanghai, China). The free-radical capacity of PJ was expressed as ascorbic acid (mM) equivalent per 100 mL of crude PJ (mM AAE/100 mL).

#### 3.6.5. Ferric Reducing Antioxidant Power (FRAP)

The antioxidant power of fruit was measured according to the method by Benzie and Strain [68] with modifications by Fawole et al. [69]. A FRAP working solution (300 mM acetate buffer (50 mL), 2,4,6-tripyridyl-s-triazine (TPTZ) (5 mL) and 20 mM FeCl_3_ (5 mL)) was freshly prepared prior to the measurements. In duplicate, 150 µL of aqueous methanolic PJ extracts (150 µL) was mixed with 2850 µL of the FRAP working solution and incubated in the dark for 30 min. Thereafter, the absorbance was measured at 593 nm using a UV–vis spectrophotometer (United scientific, SP-UV 300, Shanghai, China). The results were expressed as Trolox (mM) equivalents per 100 mL of crude PJ (mM TE/100 mL PJ).

### 3.7. Fruit Physiological Disorders and Decay

The disorders assessed included shrivelling and aril browning. These were visually inspected at 4-week intervals. The degree of incidence of the disorders was subjectively determined using a hedonic scale as described by Fawole and Opara [1], where 0 = none (no symptoms), 1 = trace (1–25%), 2 = slight (26–50%), 3 = moderate (51–75%) and 4 = severe (76–100%). The disorder index was then calculated using the following equation:

Disorder index = [(value of hedonic scale × no. of fruit at each scale number)/total no. of fruit](8)

### 3.8. Statistical Analysis

The data collected were subjected to the analysis of variance (ANOVA) using statistical software (GenStat 18th Edition, VSN International, Hemel Hempstead, UK). Mean values were separated by Duncan’s multiple range using the least significant difference (LSD) test at the 5% level of significance. Pearson’s linear correlation coefficients (r) and principal component analysis (PCA) were carried out using the statistical software XLSTAT Version 2020.4.1.1020 (Addinsoft, Paris, France).

## 4. Conclusions

Overall, the results of this study showed that the higher concentrations of EBR (CH + 10 and 15 µM EBR) had a considerable effect on the fruit quality of late-harvested pomegranate fruit. Based on the correlation and PCA analysis, CH + 10 µM EBR significantly delayed fruit metabolic changes, resulting in higher fruit texture, aril hardness, DPPH, TSS and colour intensity. This was mainly attributed to the higher DPPH and ascorbic acid of the treatment. However, the high phenolics, including the anthocyanins and FRAP of the CH + 15 µM EBR treatment, showed the best effectiveness in terms of reducing the susceptibility of late-harvested pomegranate fruit to physiological disorders, particularly aril browning and shrivelling. However, CH + 10 µM EBR treatment could be recommended to maintain quality and reduce susceptibility to physiological disorders of the late-harvested pomegranate fruit due to its extended and broader effect compared to the CH + 15 µM EBR treatment. Studies investigating the interaction of EBRs with other plant hormones, growth regulators and a combination of EBR treatment with other postharvest technologies such as controlled atmosphere, modified atmosphere and edible coating technologies are still required.

In addition, enzymatic, metabolomic and proteomics-based studies to further understand the mechanism of brassinosteroids’ exogenous postharvest application in fruit and vegetables are also still required.

## Figures and Tables

**Table 1:** Fruit firmness, aril hardness and electrolyte leakage of uncoated (control) and coated pomegranate fruit during storage at 5 °C for 12 weeks plus 3 days at 20 °C.

Parameter	Treatment	Harvest	Weeks of Cold Storage + 3 Days at 20 °C		
			4	8	12
Fruit firmness (N)		12.86 ± 0.29			
	Control		10.81 ± 0.64 ^b–f^	10.56 ± 0.48 ^b–f^	8.32 ± 0.34 ^a^
	CH 1.5%		9.77 ± 0.53 ^bc^	10.24 ± 0.48 ^b–e^	9.79 ± 0.44 ^bc^
	CH + EBR (2 µM)		10.02 ± 0.47 ^b–d^	11.5 ± 0.45 ^d–f^	9.55 ± 0.44 ^ab^
	CH + EBR (5 µM)		11.71 ± 0.42 ^ef^	11.72 ± 0.30 ^ef^	10.33 ± 0.38 ^b–e^
	CH + EBR (10 µM)		11.42 ± 0.40 ^d–f^	11.11 ± 0.45 ^c–f^	10.8 ± 0.39 ^b–f^
	CH + EBR (15 µM)		11.73 ± 0.47 ^ef^	11.93 ± 0.43 ^f^	9.91 ± 0.61 ^bc^
Aril hardness (N)		12.55 ± 0.3			
	Control		10.77 ± 0.19 ^a–d^	10.57 ± 0.62 ^a–c^	9.86 ± 0.54 ^a^
	CH 1.5%		11.48 ± 0.48 ^cd^	10.94 ± 0.64 ^a–d^	9.87 ± 0.5 ^a^
	CH + EBR (2 µM)		11.52 ± 0.30 ^cd^	10.97 ± 0.49 ^a–d^	9.94 ± 0.32 ^ab^
	CH + EBR (5 µM)		11.32 ± 0.47 ^a–d^	10.95 ± 0.45 ^a–d^	10.45 ± 0.17 ^a–c^
	CH + EBR (10 µM)		12.13 ± 0.24 ^d^	11.4 ± 0.55 ^b–d^	11.3 ± 0.57 ^a–d^
	CH + EBR (15 µM)		11.67 ± 0.24 ^cd^	11.44 ± 0.48 ^cd^	10.71 ± 0.41 ^a–c^
Electrolyte leakage (%)		3.91 ± 0.88			
	Control		7.64 ± 1.24 ^ab^	27.52 ± 2.57 ^fg^	43.15 ± 1.97 ^i^
	CH 1.5%		10.61 ± 1.97 ^bc^	15.61 ± 2.03 ^cd^	35.1 ± 2.26 ^h^
	CH + EBR (2 µM)		16.44 ± 3.76 ^cd^	27.23 ± 1.01 ^fg^	29.19 ± 3.71 ^f–h^
	CH + EBR (5 µM)		3.1 ± 0.06 ^a^	19.64 ± 2.85 ^de^	22.86 ± 1.96 ^ef^
	CH + EBR (10 µM)		5.38 ± 1.31 ^ab^	14.64 ± 0.04 ^cd^	27.54 ± 0.79 ^fg^
	CH + EBR (15 µM)		3.4 ± 0.2 ^a^	14.7 ± 0.43 ^cd^	30.46 ± 0.93 ^gh^
	Significance level (*p*)		Coating Treatment (A)	Storage period (B)	A × B
	Fruit firmness		<0.001	<0.001	0.105
	Aril hardness		0.023	<0.001	0.990
	Electrolyte leakage		<0.001	<0.001	<0.001

Data presented as mean ± SE. Different letters in each column indicate significant differences between the interaction effect of storage time and treatments at *p* < 0.05 according to Duncan’s multiple range test. SE—standard error.

**Table 2:** Titratable acidity (TA), total soluble solids (TSS) and BrimA of uncoated (control) and coated pomegranate fruit during storage at 5 °C for 12 weeks plus 3 days at 20 °C.

Parameter	Treatment	Harvest	Storage Period (Weeks of Cold Storage + 3 Days at 20 °C)		
			4	8	12
TA (% citric acid)		1.68 ± 0.24			
	Control		1.01 ± 0.14 ^a–c^	0.85 ± 0.09 ^ab^	0.81 ± 0.04 ^a^
	CH 1.5%		1.12 ± 0.10 ^a–c^	0.99 ± 0.06 ^a–c^	0.85 ± 0.04 ^ab^
	CH + EBR (2 µM)		0.96 ± 0.04 ^a–c^	0.97 ± 0.08 ^a–c^	0.97 ± 0.10 ^a–c^
	CH + EBR (5 µM)		1.19 ± 0.18 ^a^	1.05 ± 0.03 ^a–c^	0.96 ± 0.08 ^a–c^
	CH + EBR (10 µM)		1.12 ± 0.01 ^bc^	0.93 ± 0.07 ^a–c^	0.93 ± 0.08 ^a–c^
	CH + EBR (15 µM)		1.07 ± 0.06 ^ab^	0.97 ± 0.07 ^a–c^	1 ± 0.03 ^a–c^
TSS (°Brix)		18.03 ± 0.15			
	Control		17.47 ± 0.15 ^cd^	17.3 ± 0.06 ^a–c^	17.03 ± 0.07 ^a^
	CH 1.5%		17.43 ± 0.17 ^b–d^	17.37 ± 0.09 ^b–d^	17.1 ± 0.12 ^ab^
	CH + EBR (2 µM)		17.93 ± 0.03 ^e^	17.93 ± 0.67 ^ef^	17.1 ± 0.06 ^ab^
	CH + EBR (5 µM)		17.43 ± 0.03 ^b–d^	17.4 ± 0.15 ^b–d^	17.33 ± 0.03 ^a–d^
	CH + EBR (10 µM)		17.4 ± 0.06 ^b–d^	17.33 ± 0.15 ^b–d^	17.67 ± 0.09 ^de^
	CH + EBR (15 µM)		17.43 ± 0.19 ^b–d^	17.33 ± 0.12 ^b–d^	17.13 ± 0.07 ^a–c^
BrimA		15.09 ± 0.71			
	Control		15.44 ± 0.24 ^ab^	15.6 ± 0.23 ^a–d^	15.41 ± 0.07 ^a–c^
	CH 1.5%		15.22 ± 0.05 ^ab^	15.39 ± 0.16 ^ab^	15.4 ± 0.17 ^ab^
	CH + EBR (2 µM)		16.01 ± 0.1 ^cd^	16 ± 0.12 ^cd^	15.16 ± 0.26 ^a^
	CH + EBR (5 µM)		15.05 ± 0.33 ^a^	15.34 ± 0.02 ^ab^	15.4 ± 0.14 ^a–c^
	CH + EBR (10 µM)		15.17 ± 0.15 ^a^	15.54 ± 0.15 ^a–d^	15.8 ± 0.24 ^b–d^
	CH + EBR (15 µM)		15.29 ± 0.2 ^ab^	15.5 ± 0.04 ^a–d^	15.13 ± 0.14 ^a^
	Significance level (*p*)		Coating treatment (A)	Storage period (B)	A × B
	TA		0.279	0.011	0.932
	TSS		<0.001	<0.001	<0.001
	BrimA		0.038	0.131	0.042

Data presented as mean ± SE. Different letters in each column indicate significant differences between the interaction effect of storage time and treatments at *p* < 0.05 according to Duncan’s multiple range test. SE—standard error.

**Table 3:** Pearson’s correlation matrix between quality attributes of uncoated (control) and coated pomegranate fruit stored for 12 weeks at 5 °C plus 3 days at 20 °C. Values in bold are different from 0 with a significance level alpha = 0.05. Abbreviations: weight loss (WL), respiration rate (RR), fruit texture (FT), aril hardness (AH), redness (a*), colour intensity (C*), hue angle (h°), titratable acidity (TA), total soluble solids (TSS), ascorbic acid (AA), total phenolic content (TPC), total anthocyanin content (TAC), 2,2-diphenyl-1-picryl hydrazyl (DPPH) free radical scavenging activity, and ferric ion reducing antioxidant power (FRAP).

	WL	RR	FT	AH	EL	a* (Peel)	C* (Peel)	h° (Peel)	A * (Arils)	C* (Arils)	h° (Arils)	TA	TSS	BrimA	AA	TPC	TAC	DPPH	FRAP	Decay	Shrivel
WL	**1**																				
RR	0.612	**1**																			
FT	−**0.867**	−0.774	**1**																		
AH	−**0.941**	−0.456	0.769	**1**																	
EL	0.749	0.496	−**0.860**	−0.566	**1**																
a* (Peel)	0.016	−0.406	0.196	−0.291	−0.357	**1**															
C* (Peel)	−0.016	−0.429	0.206	−0.270	−0.368	**0.998**	**1**														
h° (Peel)	−0.225	−0.501	0.117	−0.053	−0.188	0.614	0.665	**1**													
a* (Arils)	−0.515	−0.092	0.613	0.673	−0.489	−0.373	−0.403	−0.690	**1**												
C* (Arils)	−0.606	−0.221	0.664	0.775	−0.446	−0.407	−0.429	−0.612	**0.976**	**1**											
h° (Arils)	−0.196	−0.533	0.016	0.166	0.316	0.019	0.063	0.610	−0.477	−0.275	**1**										
TA	−0.743	−0.416	0.611	0.508	−**0.814**	0.438	0.478	0.603	0.050	0.054	0.027	**1**									
TSS	−0.789	−0.428	0.804	**0.872**	−0.596	−0.418	−0.413	−0.328	**0.843**	**0.895**	−0.080	0.281	**1**								
BrimA	−0.314	−0.166	0.409	0.541	−0.076	−0.682	−0.700	−0.688	0.788	**0.839**	−0.086	−0.344	0.805	**1**							
AA	−0.639	−0.688	0.661	0.695	−0.273	0.104	0.101	0.029	0.462	0.610	0.413	0.178	0.535	0.416	**1**						
TPC	−0.679	−0.498	0.777	0.438	−**0.974**	0.466	0.485	0.369	0.283	0.236	−0.248	**0.875**	0.442	−0.111	0.145	**1**					
TAC	−0.628	−0.356	0.673	0.386	−**0.945**	0.467	0.487	0.373	0.230	0.163	−0.316	**0.906**	0.350	−0.221	0.039	**0.984**	**1**				
DPPH	−**0.915**	−0.605	0.674	**0.911**	−0.431	−0.142	−0.102	0.307	0.320	0.478	0.528	0.572	0.662	0.299	0.716	0.372	0.317	**1**			
FRAP	−0.666	−0.374	0.739	0.468	−**0.954**	0.509	0.515	0.241	0.391	0.330	−0.368	**0.856**	0.400	−0.142	0.246	**0.942**	**0.953**	0.352	**1**		
Decay	0.695	0.673	−**0.865**	−0.477	**0.930**	−0.353	−0.374	−0.327	−0.330	−0.318	0.111	−0.737	−0.580	−0.111	−0.232	−**0.941**	−**0.870**	−0.421	−0.807	**1**	
Shrivel	0.770	0.448	−0.730	−0.568	**0.896**	−0.502	−0.522	−0.414	−0.277	−0.265	0.129	−**0.944**	−0.376	0.218	−0.351	−**0.902**	−**0.917**	−0.543	−**0.959**	**0.759**	**1**

## Data Availability

The data presented in this study are available on request from the corresponding author. The data is not publicly available due to an ongoing PhD program.

## References

[1] Fawole O.A., Opara U.L. (2013). Effects’ of storage temperature and duration on physiological responses of pomegranate fruit. Ind. Crops Prod..

[2] Opara I.K., Fawole O.A., Opara U.L. (2021). Postharvest Losses of Pomegranate Fruit at the Packhouse and Implications for Sustainability Indicators. Sustainability.

[3] Kahramanoglu I. (2019). Trends in pomegranate sector: Production, postharvest handling and marketing. IJAFLS Int. J. Agric. For. Life Sci..

[4] Opara U.L., Atukuri J., Fawole O.A. (2015). Application of physical and chemical postharvest treatments to enhance storage and shelf life of pomegranate fruit—A review. Sci. Hortic..

[5] Fawole O.A., Opara U.L. (2013). Harvest discrimination of pomegranate fruit: Postharvest quality changes and relationships between instrumental and sensory attributes during shelf life. J. Food Sci..

[6] Defilippi B.G., Whitaker B.D., Hess-Pierce B.M., Kader A.A. (2006). Development and control of scald on wonderful pomegranates during long-term storage. Postharvest Biol. Technol..

[7] Kashash Y., Doron-Faigenboim A., Holland D., Porat R. (2019). Effects of harvest time on chilling tolerance and the transcriptome of ‘Wonderful’ pomegranate fruit. Postharvest Biol. Technol..

[8] Adetunji C.O., Fawole O.B., Arowora K.A., Nwaubani S.I., Ajayi E.S., Oloke J.K., Majolagbe O.M., Ogundele B.A., Aina J.A., Adetunji J.B. (2012). Effects of edible coatings from Aloe vera gel on quality and postharvest physiology of *Ananas comosus* L. fruit during ambient storage. Glob. J. Sci. Front. Res. Bio-Tech Genet..

[9] Antunes M.D., Gago C.M., Cavaco A.M., Miguel M.G. (2012). Edible coatings enriched with essential oils and their compounds for fresh and fresh-cut fruit. Recent Pat. Food Nutr. Agric..

[10] Baldwin E., Burns J., Kazokas W., Brecht J., Hagenmaier R., Bender R., Pesis E. (1999). Effect of two edible coatings with different permeability characteristics on mango (*Mangifera indica* L.) ripening during storage. Postharvest Biol. Technol..

[11] Dong H., Cheng L., Tan J., Zheng K., Jiang Y. (2004). Effects of chitosan coating on quality and shelf life of peeled litchi fruit. J. Food Eng..

[12] Ehteshami S., Abdollahi F., Ramezanian A., Dastjerdi A.M., Rahimzadeh M. (2019). Enhanced chilling tolerance of pomegranate fruit by edible coatings combined with malic and oxalic acid treatments. Sci. Hortic..

[13] Fawole O.A., Riva S.C., Opara U.L. (2020). Efficacy of Edible Coatings in Alleviating Shrivel and Maintaining Quality of Japanese Plum (*Prunus salicina* Lindl.) during Export and Shelf Life Conditions. Agronomy.

[14] Kawhena T.G., Tsige A.A., Opara U.L., Fawole O.A. (2020). Application of Gum Arabic and Methyl Cellulose Coatings Enriched with Thyme Oil to Maintain Quality and Extend Shelf Life of “Acco” Pomegranate Arils. Plants.

[15] Kerch G. (2015). Chitosan films and coatings prevent losses of fresh fruit nutritional quality: A review. Trends Food Sci. Technol..

[16] Arroyo B.J., Bezerra A.C., Oliveira L.L., Arroyo S.J., Melo E.A., Santos A.M.P. (2020). Antimicrobial active edible coating of alginate and chitosan add ZnO nanoparticles applied in guavas (*Psidium guajava* L.). Food Chem..

[17] Fawole O.A., Atukuri J., Arendse E., Opara U.O. (2020). Postharvest physiological responses of pomegranate fruit (cv. Wonderful) to exogenous putrescine treatment and effects on physico-chemical and phytochemical properties. Food Sci. Hum..

[18] Nisperos-Carriedo M.O., Baldwin E.A., Shaw P.E. (1992). Development of an edible coating for extending postharvest life of selected fruits and vegetables. Proceedings of the Florida State Horticultural Society Meeting.

[19] Riva S.C., Opara U.O., Fawole O.A. (2020). Recent developments on postharvest application of edible coatings on stone fruit: A review. Sci. Hortic..

[20] Tavassoli-Kafrani E., Shekarchizadeh H., Masoudpour-Behabadi M. (2016). Development of edible films and coatings from alginates and carrageenans. Carbohydr. Polym..

[21] Tesfay S.Z., Magwaza L.S. (2017). Evaluating the efficacy of moringa leaf extract, chitosan and carboxymethyl cellulose as edible coatings for enhancing quality and extending postharvest life of avocado (*Persea americana* Mill.) fruit. Food Packag. Shelf Life.

[22] Jiao W.X., Shu C., Li X.X., Cao J.K., Fan X.G., Jiang W.B. (2019). Preparation of a chitosan-chlorogenic acid conjugate and its application as edible coating in postharvest preservation of peach fruit. Postharvest Biol. Technol..

[23] Shen Y., Yang H. (2017). Effect of preharvest chitosan- g -salicylic acid treatment on postharvest table grape quality, shelf life, and resistance to Botrytis cinerea -induced spoilage. Sci. Hortic..

[24] Cazón P., Velazquez G., Ramírez J.A., Vázquez M. (2017). Polysaccharide-based films and coatings for food packaging: A review. Food Hydrocoll..

[25] Meng X.H., Qin G.Z., Tian S.P. (2010). Influences of preharvest spraying Cryptococcus laurentii combined with postharvest chitosan coating on postharvest diseases and quality of table grapes in storage. Lwt-Food Sci Technol..

[26] Kumari P., Barman K., Patel V.B., Siddiqui M.W., Kole B. (2015). Reducing postharvest pericarp browning and preserving health promoting compounds of litchi fruit by combination treatment of salicylic acid and chitosan. Sci. Hortic..

[27] Coll Y., Coll F., Amorós A., Pujol M. (2015). Brassinosteroids roles and applications: An up-date. Biologia.

[28] Ali B. (2017). Practical applications of brassinosteroids in horticulture-Some field perspectives. Sci. Hortic..

[29] Bajguz A., Hayat S. (2009). Effects of brassinosteroids on the plant responses to environmental stresses. Plant Physiol. Biochem..

[30] Hussain M.A., Fahad S., Sharif R., Jan M.F., Mujtaba M., Ali Q., Ahmad A., Ahmad H., Amin N., Ajayo B.S. (2020). Multifunctional role of brassinosteroid and its analogues in plants. Plant Growth Regul..

[31] Baghel M., Nagaraja A., Srivastav M., Meena N.K., Kumar M.S., Kumar A., Sharma R.R. (2019). Pleiotropic influences of brassinosteroids on fruit crops: A review. Plant Growth Regul..

[32] EFSA (2020). Peer review of the pesticide risk assessment of the activesubstance 24-epibrassinolide. EFSA J..

[33] EPA (2019). 24-Epibrassinolide; Exemption from the Requirement of a Tolerance. Fed. Regist..

[34] Wang Q., Ding T., Gao L., Pang J., Yang N. (2012). Effect of brassinolide on chilling injury of green bell pepper in storage. Sci. Hortic..

[35] Wang X., Lu Z., Su J., Li Y., Cao M., Gao H. (2020). 24-Epibrassinolide delays senescence in harvested kiwifruit through effects on mitochondrial membrane and antioxidant activity. LWT.

[36] Habibi F., Serrano M., Zacarias L., Valero D., Guillen F. (2021). Postharvest Application of 24-Epibrassinolide Reduces Chilling Injury Symptoms and Enhances Bioactive Compounds Content and Antioxidant Activity of Blood Orange Fruit. Front Plant Sci..

[37] Aghdam M.S., Mohammadkhani N. (2013). Enhancement of Chilling Stress Tolerance of Tomato Fruit by Postharvest Brassinolide Treatment. Food Bioprocess Technol..

[38] Gao H., Kang L., Liu Q., Cheng N., Wang B., Cao W. (2015). Effect of 24-epibrassinolide treatment on the metabolism of eggplant fruits in relation to development of pulp browning under chilling stress. J. Food Sci. Technol..

[39] Gao H., Zhang Z., Lv X., Cheng N., Peng B., Cao W. (2016). Effect of 24-epibrassinolide on chilling injury of peach fruit in relation to phenolic and proline metabolisms. Postharvest Biol. Technol..

[40] Aghdam M.S., Asghari M., Farmani B., Mohayeji M., Moradbeygi H. (2012). Impact of postharvest brassinosteroids treatment on PAL activity in tomato fruit in response to chilling stress. Sci. Hortic..

[41] Ghorbani B., Pakkish Z. (2014). Brassinosteroid Enhances Cold Stress Tolerance of Washington Navel Orange (*Citrus sinensis* L.) Fruit by Regulating Antioxidant Enzymes during Storage. Agric. Conspec. Sci..

[42] Pakkish Z., Ghorbani B., Najafzadeh R. (2019). Fruit quality and shelf life improvement of grape cv. Rish Baba using Brassinosteroid during cold storage. J. Food Meas. Charact..

[43] Liu Z., Li L., Luo Z., Zeng F., Jiang L., Tang K. (2016). Effect of brassinolide on energy status and proline metabolism in postharvest bamboo shoot during chilling stress. Postharvest Biol. Technol..

[44] Li T., Yun Z., Wu Q., Zhang Z., Liu S., Shi X., Duan X., Jiang Y. (2018). Proteomic profiling of 24-epibrassinolide-induced chilling tolerance in harvested banana fruit. J. Proteom..

[45] Zhang Y., Zhang M., Yang H. (2015). Postharvest chitosan-g-salicylic acid application alleviates chilling injury and preserves cucumber fruit quality during cold storage. Food Chem..

[46] Sayyari M., Aghdam M.S., Salehi F., Ghanbari F. (2016). Salicyloyl chitosan alleviates chilling injury and maintains antioxidant capacity of pomegranate fruits during cold storage. Sci. Hortic..

[47] Wu L., Yang H. (2016). Combined Application of Carboxymethyl Chitosan Coating and Brassinolide Maintains the Postharvest Quality and Shelf Life of Green Asparagus. J. Food Process. Preserv..

[48] Zhu Z., Zhang Z., Qin G., Tian S. (2010). Effects of brassinosteroids on postharvest disease and senescence of jujube fruit in storage. Postharvest Biol. Technol..

[49] Baswal A.K., Dhaliwal H.S., Singh Z., Mahajan B.V.C., Gill K.S. (2020). Postharvest application of methyl jasmonate, 1-methylcyclopropene and salicylic acid extends the cold storage life and maintain the quality of ‘Kinnow’ mandarin (*Citrus* nobilis L. X C. *deliciosa* L.) fruit. Postharvest Biol. Technol..

[50] Sayyari M., Ghanbari F. (2013). Effect of acetyl salicylic acid on quality and chilling resistance of sweet pepper (*Capsicum annuum* L.) at different storage temperatures. Acta Hortic..

[51] Ncama K., Magwaza L.S., Fawole O.A., Tesfay S.Z., Opara U.L. (2018). Investigating pre-symptomatic biochemical markers related to ‘Marsh’ grapefruit (Citrus × paradisi Macfad) susceptibility to chilling injury and rind pitting disorders. Acta Hortic..

[52] Sayyari M., Babalar M., Kalantari S., Martínez-Romero D., Guillén F., Serrano M., Valero D. (2011). Vapour treatments with methyl salicylate or methyl jasmonate alleviated chilling injury and enhanced antioxidant potential during postharvest storage of pomegranates. Food Chem..

[53] Nazoori F., ZamaniBahramabadi E., Mirdehghan S.H., Rafie A. (2020). Extending the shelf life of pomegranate (*Punica granatum* L.) by GABA coating application. J. Food Meas. Charact.

[54] Arendse E., Fawole O.A., Opara U.L. (2014). Influence of storage temperature and duration on postharvest physico-chemical and mechanical properties of pomegranate fruit and arils. CyTA J. Food.

[55] Mditshwa A., Magwaza L.S., Tesfay S.Z., Opara U.L. (2017). Postharvest factors affecting vitamin C content of citrus fruits: A review. Sci. Hortic..

[56] Meighani H., Ghasemnezhad M., Bakhshi D. (2015). Effect of different coatings on post-harvest quality and bioactive compounds of pomegranate (*Punica granatum* L.) fruits. J. Food Sci. Technol..

[57] Zhang W., Jing L., Chen H., Zhang S. (2021). NC-1 coating combined with 1-MCP treatment maintains better fruit qualities in honey peach during low-temperature storage. Int. J. Food Sci. Tech..

[58] Sinha A., Gill P.P.S., Jawandha S.K., Kaur P., Grewal S.K. (2021). Chitosan-enriched salicylic acid coatings preserves antioxidant properties and alleviates internal browning of pear fruit under cold storage and supermarket conditions. Postharvest Biol. Technol..

[59] Hashemi M., Dastjerdi A.M., Mirdehghan S.H., Shakerardekani A., Golding J.B. (2021). Incorporation of Zataria multiflora Boiss essential oil into gum Arabic edible coating to maintain the quality properties of fresh in-hull pistachio (*Pistacia vera* L.). Food Packag. Shelf Life.

[60] Tahir H.E., Xiaobo Z., Jiyong S., Mahunu G.K., Zhai X., Mariod A.A. (2018). Quality and postharvest-shelf life of cold-stored strawberry fruit as affected by gum arabic (*Acacia senegal*) edible coating. J. Food Biochem..

[61] Hanif A., Ahmad S., Shahzad S., Liaquat M., Anwar R. (2020). Postharvest application of salicylic acid reduced decay and enhanced storage life of papaya fruit during cold storage. J. Food Meas. Charact..

[62] Zhu F., Yun Z., Ma Q., Gong Q., Zeng Y., Xu J., Cheng Y., Deng X. (2015). Effects of exogenous 24-epibrassinolide treatment on postharvest quality and resistance of Satsuma mandarin (*Citrus unshiu*). Postharvest Biol. Technol..

[63] Ding Y., Zhu Z., Zhao J., Nie Y., Zhang Y., Sheng J., Meng D., Mao H., Tang X. (2016). Effects of Postharvest Brassinolide Treatment on the Metabolism of White Button Mushroom (*Agaricus bisporus*) in Relation to Development of Browning During Storage. Food Bioproc. Tech..

[64] Mirdehghan S.H., Rahemi M., Castillo S., Martínez-Romero D., Serrano M., Valero D. (2007). Pre-storage application of polyamines by pressure or immersion improves shelf-life of pomegranate stored at chilling temperature by increasing endogenous polyamine levels. Postharvest Biol. Technol..

[65] Sayyari M., Valero D., Babalar M., Kalantari S., Zapata P.J., Serrano M. (2010). Prestorage oxalic acid treatment maintained visual quality, bioactive compounds, and antioxidant potential of pomegranate after long-term storage at 2 degrees C. J. Agric. Food Chem..

[66] Fawole O.A., Opara U.L. (2013). Fruit growth dynamics, respiration rate and physico-textural properties during pomegranate development and ripening. Sci. Hortic..

[67] Fawole O.A., Opara U.L. (2013). Changes in physical properties, chemical and elemental composition and antioxidant capacity of pomegranate (cv. Ruby) fruit at five maturity stages. Sci. Hortic..

[68] Benzie I.F., Strain J.J. (1996). The ferric reducing ability of plasma (FRAP) as a measure of “antioxidant power”: The FRAP assay. Anal. Biochem..

[69] Fawole O.A., Makunga N.P., Opara U.L. (2012). Antibacterial, antioxidant and tyrosinase-inhibition activities of pomegranate fruit peel methanolic extract. BMC Complement. Altern. Med..

